# *Serpine1* Knockdown Enhances MMP Activity after Flexor Tendon Injury in Mice: Implications for Adhesions Therapy

**DOI:** 10.1038/s41598-018-24144-1

**Published:** 2018-04-11

**Authors:** Margaret A. T. Freeberg, Youssef M. Farhat, Anas Easa, Jacob G. Kallenbach, Dominic W. Malcolm, Mark R. Buckley, Danielle S. W. Benoit, Hani A. Awad

**Affiliations:** 1 Department of Biomedical Engineering, University of Rochester, Rochester, NY USA; 2 Center for Musculoskeletal Research, University of Rochester Medical Center, Rochester, NY USA; 3 Department of Orthopedics, University of Rochester Medical Center, Rochester, NY USA

## Abstract

Injuries to flexor tendons can be complicated by fibrotic adhesions, which severely impair the function of the hand. Adhesions have been associated with TGF-β1, which causes upregulation of PAI-1, a master suppressor of protease activity, including matrix metalloproteinases (MMP). In the present study, the effects of inhibiting PAI-1 in murine zone II flexor tendon injury were evaluated utilizing knockout (KO) mice and local nanoparticle-mediated siRNA delivery. In the PAI-1 KO murine model, reduced adherence of injured tendon to surrounding subcutaneous tissue and accelerated recovery of normal biomechanical properties compared to wild type controls were observed. Furthermore, MMP activity was significantly increased in the injured tendons of the PAI-1 KO mice, which could explain their reduced adhesions and accelerated remodeling. These data demonstrate that PAI-1 mediates fibrotic adhesions in injured flexor tendons by suppressing MMP activity. *In vitro* siRNA delivery to silence *Serpine1* expression after treatment with TGF-β1 increased MMP activity. Nanoparticle-mediated delivery of siRNA targeting *Serpine1* in injured flexor tendons significantly reduced target gene expression and subsequently increased MMP activity. Collectively, the data demonstrate that PAI-1 can be a druggable target for treating adhesions and accelerating the remodeling of flexor tendon injuries.

## Introduction

Flexor tendon injuries in zone II of the hand are prone to the formation of debilitating adhesions, resulting from fibrotic scar tissue, which obstructs tendon gliding and severely impairs flexion of the injured digits and the function of the afflicted hand. These injuries affect over 140,000 full-time employees in the United States alone, losing over one million workdays annually^1^. Furthermore, adhesion formation is not limited to hand surgery, but is considered a substantial postoperative complication in all surgical procedures. While the incidence and severity of postoperative adhesions vary between surgical specialties^2^, identifying the mechanisms of adhesions and designing novel pharmacological or biological therapies to mitigate them remain as unmet clinical needs.

Adhesions in tendon^3,4^ as well as throughout the body have been causatively associated with Transforming Growth Factor Beta 1 (TGF-β1)^5,6^. TGF-β1 stimulates myofibroblast activation, leading to increased matrix synthesis and decreased matrix remodeling^7^. Studies on the healing of flexor digitorum profundus (FDP) tendon in chicken demonstrated that TGF-β1 is highly expressed throughout the early healing period, particularly in the peritendinous region, as evidenced by immunohistochemistry^8^. Thus, TGF-β1 has been identified as a therapeutic target for adhesions. Neutralizing antibodies to TGF-β1 have been shown to reduce rat flexor tendon adhesions following transection^3^, and TGF-β1 inhibitors such as mannose-6-phosphate also improve postoperative range of motion (ROM) in rabbit zone II flexor tendons^9^. These treatments, however, reduce tendon mechanical strength, especially at higher doses^3,9^. We have recently demonstrated that disruption of canonical TGF-β1 signaling in Smad3^−/−^ mice reduced flexor tendon adhesions and improved tendon gliding, due to reduced extracellular matrix (ECM) deposition, but led to compromised biomechanical properties with a significant reduction in repair strength^4^. Thus, the indispensable reparative effects of TGF-β1 limit the applicability of TGF-β1-directed therapeutics in load bearing tendons. In lieu of this approach, we hypothesized that canonical TGF-β1 signaling induces downstream mediators of fibrosis, which may include novel therapeutic targets. One such target is the protease suppressor, plasminogen activator inhibitor 1 or PAI-1.

Activation of canonical TGF-β1 directly upregulates PAI-1^10,11^. As a direct inhibitor of both tissue- and urokinase-type plasminogen activators (tPA and uPA, respectively), PAI-1 acts as a master regulator of plasmin activity, thereby regulating fibrinolysis and plasmin-mediated activation of matrix metalloproteinases (MMP)^11^. As a repressor of MMP activity, increased PAI-1 has been implicated in major organ fibrotic conditions including skin, liver, kidney, and lung^12–18^ and strategies to abrogate its effects have demonstrated preclinical proof of efficacy^19,20^. However, formal evaluation of the role of PAI-1 in tendon adhesions following injury has not been previously explored.

The objective of this study was to assess effects of PAI-1 therapeutic inhibition on flexor tendon healing using a zone II injury to the deep digital flexor tendon in mice. We first utilized a PAI-1 knockout (PAI-1 KO) mouse to evaluate the effects of global gene deletion of *Serpine1* (which encodes for PAI-1) on flexor tendon healing over time compared to C57Bl6/J (WT) controls. Next, we used siRNA approaches to knockdown *Serpine1* to test whether this approach reverses PAI-1 suppression of protease activity in tendon fibroblast cultures *in vitro*. Finally, we treated flexor tendon injuries in WT mice using a nanoparticle-mediated localized siRNA delivery strategy^21,22^ to inhibit *Serpine1* gene expression *in vivo* and evaluate repair tissue remodeling.

## Results

### PAI-1 is abundant in fibrotic tendon tissue

**Figure 1:**
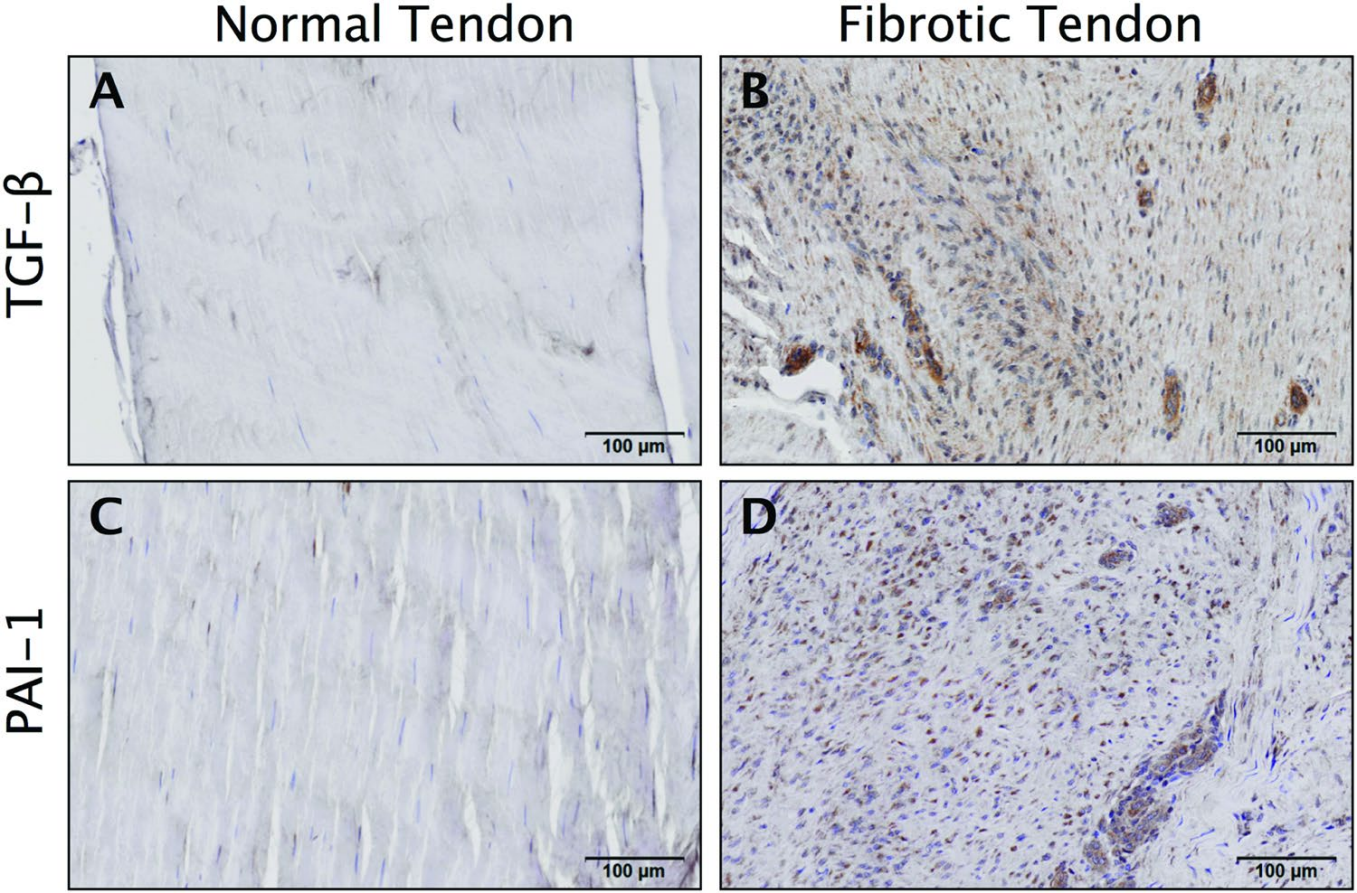
TGF-β1 and PAI-1 are ubiquitous in fibrotic human tendon tissue. Immunohistochemical (IHC) staining of normal human tendon (**A** and **C**) and fibrotic Dupuytren’s contracture tissue (**B** and **D**) against TGF-β1 and PAI-1. Brown staining in B and D indicates increased TGF-β1 and PAI-1, respectively.

**Figure 2:**
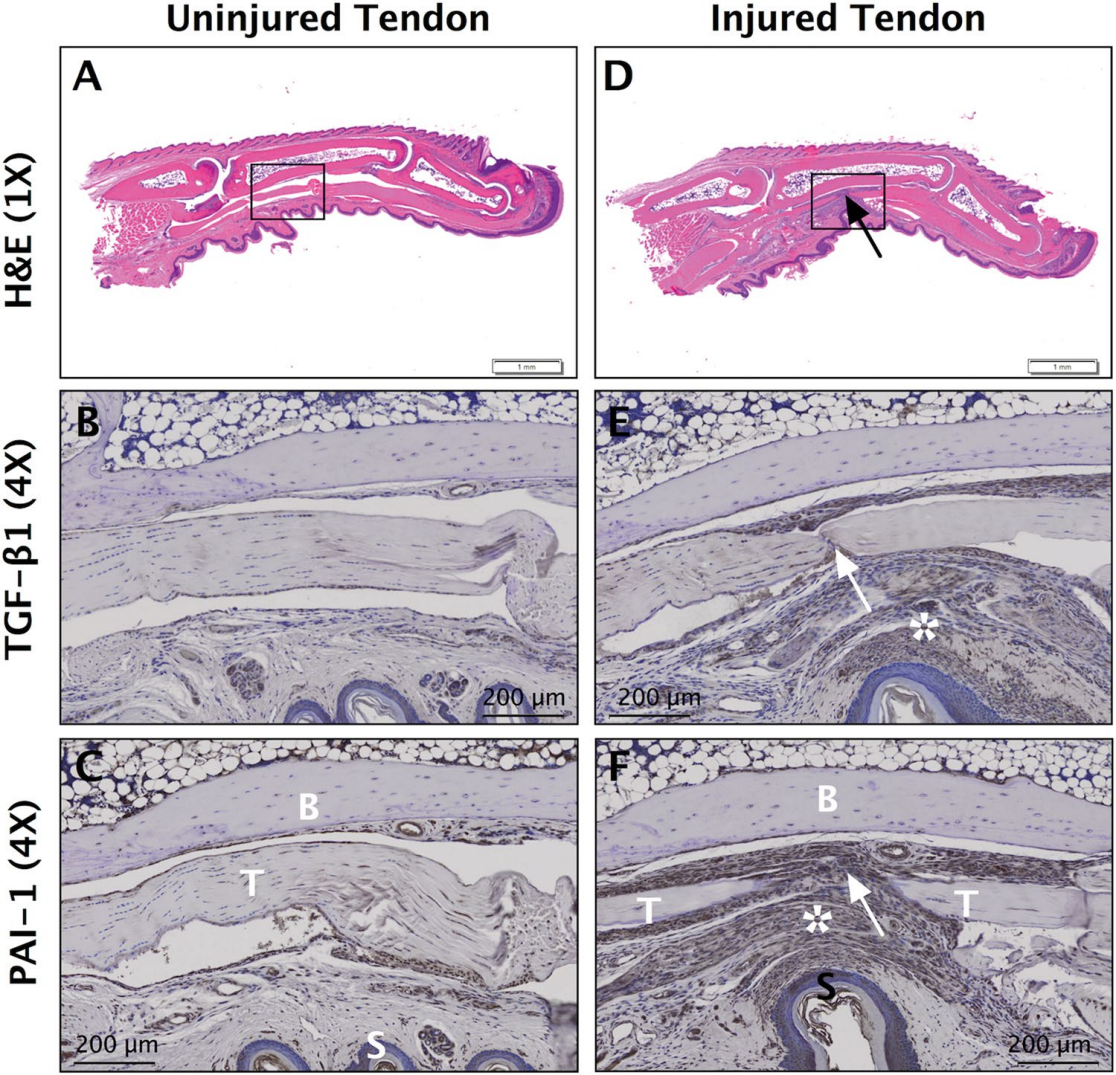
TGF-β1 and PAI-1 are abundant in injured mouse flexor tendon adhesions. Sagittal sections of uninjured (**A**–**C**) and Zone II partially-lacerated (**D**–**F**) deep digital flexor tendons at 14 dpi. Sections are stained using hematoxylin and eosin (**E** and **H**) and antibodies against TGF-β1 and PAI-1. Arrows indicate the laceration site. Box in D shows the adhesions surrounding the injured tendon (magnified in **E** and **F**). Asterisks in E and F highlight the intense brown staining indicative of increased TGF-β1 and PAI-1, respectively, in the adhesions (scar tissue). B = bone, T = tendon, and S = skin.

We sought to first determine the abundance of TGF-β1 and PAI-1 in tendon fibrosis. To that end, we performed immunohistochemistry staining on fibrotic Dupuytren’s tissue from human flexor tendons and observed ubiquitous levels of TGF-β1 and PAI-1 (Fig. 1), which is consistent with published reports implicating this pathway in mediating the fibrotic traits of Dupuytren’s disease in tendon^23^. We also observed that TGF-β1 and PAI-1 are abundant in the healing and adhesion tissue developed 14 days following zone II injury to the deep digital flexor tendon in the hind paw of C57Bl/6 J WT mice (Fig. 2).

### PAI-1 deficiency reduces healing tendon adhesions to subcutaneous tissues

**Figure 3:**
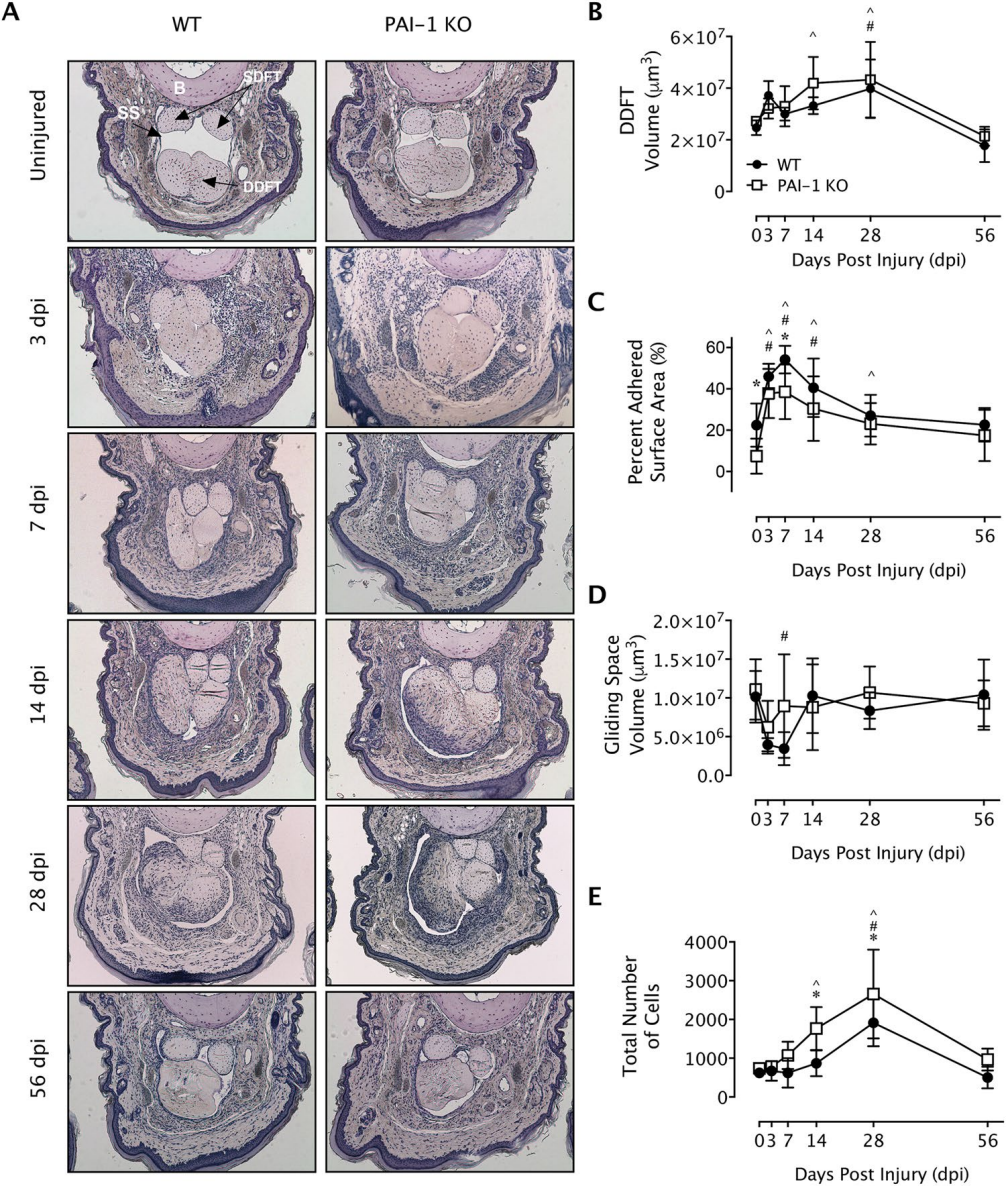
Loss of PAI-1 significantly decreased the adhesion of flexor tendons to subcutaneous tissue at early time points following flexor tendon injury in zone II of the mouse hind paw. (**A**) Representative micrographs of hematoxylin-stained cross-sections of the digits of WT (C57Bl/6 J) and PAI-1 KO mice before and after zone II injury up to 8 weeks post injury. All representative micrographs represent the third out of five levels analyzed, corresponding to the center of the region of interest. Qualitatively, thickening of the synovial sheath and increased peritendinous cellular activity (indicated by intense hematoxylin staining) were observed at early time points with manifest adhesions peaking around 7 dpi. The deep digital flexor tendons of WT and PAI-1 KO mice became highly cellular by 14 days and progressively remodeled overtime thereafter. (**B**) Histomorphometric quantification of tendon cross section area, averaged over the five levels, shows no significant differences between WT and PAI-1 KO mice. (**C**) The length of tendon perimeter adhered to the subcutaneous tissue was assessed for each of the five levels, and the total surface area of tendon adhered to the subcutaneous tissue was determined by integrating the length of adhered tendon over the five levels. The percentage of the tendon surface area adhered to subcutaneous tissues was then quantified and demonstrated significantly reduced adhesions in the PAI-1 KO mice compared to WT mice at 3 and 7 dpi. (**D**) The total volume of the sheath space around the flexor tendons, calculated by integrating the sheath space areal measurements from the five levels, significantly decreased between 3 and 7 dpi in both WT and PAI-1 KO mice. (**E**) Cells within the uninjured and injured tendons were counted in DAPI-stained sections from all five levels. Total cell counts significantly increased over time in both WT and PAI-KO mice peaking by 28 dpi. However, total cell counts in the PAI-1 KO tendons were significantly greater than the WT at 14 and 28 dpi. Data in **B–E** are expressed as mean ± standard deviation (SD). Asterisks (*) indicate significant differences between WT and PAI-1 KO (p < 0.05). Pound symbols (^#^) indicate significant differences between uninjured and injured in WT (p < 0.05). Caret symbols (^) indicate significant differences between uninjured and injured in PAI-1 KO (p < 0.05). B = proximal phalanx bone, SS = synovial sheath, SDFT = superficial digital flexor tendon, DDFT = deep digital flexor tendon, SCT = subcutaneous tissue.

The effects of gene deletion of *Serpine1* on flexor tendon adhesions were evaluated histomorphometrically over time up to 56 days post injury (dpi) in WT and PAI-1 KO mice. The flexor tendons of the middle digits of the hind paws in both murine strains were partially lacerated between the metatarsophalangeal and the proximal interphalangeal joints to simulate a zone II flexor tendon injury in the human hand (Supplementary Figure S1). To reduce dehydration-related shrinkage and to enable a more accurate histomorphometric quantification, we used a water-based mounting medium to coverslip hematoxylin-stained axial sections, allowing for excellent preservation of tissue morphology as well as sufficient contrast to distinguish between normal and injured tendons, pulleys, and subcutaneous tissues (Supplementary Figure S2). Qualitative assessment of hematoxylin-stained axial tendon sections (Fig. 3A) in both mouse strains demonstrated a thickening of the synovial sheath with increased cellularity in the extratendinous region surrounding the tendon as early as 3 and 7 dpi. Significant adhesions that compromised the gliding space and obscured the margins between the tendon and extratendinous tissues were observed by 7 dpi. In the proliferative/granulation phases of healing between 14 and 28 dpi, increased cell numbers and initial matrix deposition were observed in the healing tendon in both mouse strains. Between 28 and 56 dpi, the healing tendon began to undergo contraction and remodeling and the gliding space between the tendon and subcutaneous tissues exhibited signs of recovery in both mouse strains. A quantitative histomorphometric assessment of flexor tendon size, adhesions, sheath space and tendon cellularity was carried out systematically over 5 axial section levels spanning 500 microns encompassing the injury site (Supplementary Figure S3). Compared to uninjured tendons, the average volume of the healing tendons significantly increased in WT and PAI-1 KO digits by 28 dpi before returning to uninjured tendon size by 56 dpi. However, there were no significant differences attributed to PAI-1 deficiency (Fig. 3B). The percentage of the tendon surface area adhered to subcutaneous tissues significantly increased between 3 dpi and 7 dpi in both WT and PAI-1 KO mice, then decreased over time (Fig. 3C). Furthermore, PAI-1 deficiency significantly reduced peak adhesions compared to WT. Gliding space volume decreased significantly over time up to 7 dpi in both WT and PAI-1 KO mice before returning to uninjured levels, but there were no significant differences attributed to PAI-1 deficiency (Fig. 3D). Cell numbers in the injured tendons were quantified by counting the DAPI stained nuclei to assess the cellular activity of the healing tissue. Tendon cell counts were significantly increased over time following injury up to 28 dpi in both WT and PAI-1 KO mice before returning to uninjured levels of cellularity by 56 dpi (Fig. 3E). Furthermore, PAI-1 deficiency significantly increased tendon cellularity compared to WT at 14 and 28 dpi.

### PAI-1 deficiency accelerates injured tendon biomechanical property recovery

The functional properties of injured tendons were determined using biomechanical testing, which involved a 10-minute stress relaxation at 5% sub-failure tensile strain followed by displacement-controlled stretching of the tendon to failure at 0.05 mm/sec. The sub-failure viscoelastic properties were determined by fitting the data to a quasi-linear viscoelasticity (QLV) model, which decouples the stress response into a nonlinear elastic loading response and a time-dependent viscoelastic relaxation function (Supplementary Figure S4)^24^. In general, the injury had more significant effects on the nonlinear elastic loading response in WT tendons, but not in PAI-1 KO tendons. At 28 and 56 dpi, the peak stress at the end of the nonlinear elastic loading phase was significantly lower in injured WT tendon than the uninjured counterparts (p < 0.05). However, PAI-1 deficiency had no significant effects on the peak stress (Table 1). For WT tendons at 28 and 56 dpi, the initial slope of the nonlinear elastic stress-strain curve (parameter A × B) was significantly lower than the uninjured counterparts. Furthermore, parameter A × B was unaffected by PAI-1 deficiency in uninjured tendons but was significantly increased in PAI-1 KO compared to WT tendons at 56 dpi. The rate of change of the slope of the stress-strain curve (parameter B) significantly increased after injury in WT tendon but was unchanged throughout the healing time course in PAI-1 KO tendons. Moreover, while parameter B was lower in uninjured WT tendons compared to PAI-1 KO tendons, it was significantly increased in WT tendons at 56 dpi.

**Table 1:** Tensile Stress-Relaxation and Displacement-Controlled Tensile Failure Biomechanical Properties (mean ± SD).

	C57Bl6/J (WT)				PAI-1 KO			
	Uninjured	14 dpi	28 dpi	56 dpi	Uninjured	14 dpi	28 dpi	56 dpi
**Quasi-Linear Viscoelastic (QLV) Properties**								
Non-Linear Elastic Stress-Strain Loading Response								
Peak Stress [MPa]	8.73 ± 3.21	5.86 ± 2.32	4.63 ± 1.89^b^	4.97 ± 1.80^b^	7.11 ± 5.16	6.58 ± 4.49	7.54 ± 2.27	5.49 ± 1.32
Initial Stress-Strain Slope (A×B) [MPa]	182.1 ± 100.0	69.9 ± 65.3	92.5 ± 49.2	40.4 ± 28.7^b^	125.2 ± 137.3	140.8 ± 125.0	164.6 ± 107.3	177.9 ± 164^a^
Stress-Strain Rate of Change (B)	24.1 ± 23.1	48.7 ± 11.6^b^	40.1 ± 12.7	72.2 ± 16.5^b^	49.7 ± 24.9^a^	38.7 ± 21.1	40.8 ± 21.7	43.2 ± 25.6^a^
Viscoelastic Stress Relaxation Response								
Stress Relaxation [%]	38.1 ± 7.12	48.1 ± 8.58^b^	43.4 ± 6.86	44.8 ± 7.27	42.2 ± 7.25	38.8 ± 7.50^a^	38.43 ± 9.51	46.8 ± 6.80
Viscous Dissipation (C)	0.28 ± 0.13	0.35 ± 0.15	0.23 ± 0.07	0.26 ± 0.08	0.26 ± 0.06	0.26 ± 0.06	0.18 ± 0.05	0.28 ± 0.10
Early Relaxation Time Constant (τ_1_) [sec]	4.18 ± 4.5	1.98 ± 1.8^b^	0.72 ± 1.1^b^	0.12 ± 0.25^b^	2.60 ± 1.1	1.46 ± 1.3	1.29 ± 1.5	0.56 ± 0.88
Late Relaxation Time Constant (τ_2_) [sec]	142.5 ± 101.8	109.4 ± 51.5	260.5 ± 231.9	165.4 ± 80.5	177.6 ± 125.4	103.1 ± 72.2	153.1 ± 52.8	176.5 ± 64.3
**Structural Properties**								
Maximum Force (N)	3.36 ± 0.60	2.26 ± 0.47^b^	1.54 ± 0.66^b^	2.54 ± 0.39	3.19 ± 0.93	2.36 ± 0.97^b^	2.66 ± 0.37^a^	2.52 ± 0.78
Stiffness (N/mm)	3.70 ± 1.00	2.51 ± 0.34^b^	1.97 ± 0.61^b^	2.98 ± 0.71^b^	4.04 ± 0.66	2.90 ± 0.84^b^	3.20 ± 0.52^a,b^	2.92 ± 1.07^b^
Work to Failure (N.mm)	2.15 ± 0.53	1.19 ± 0.30^b^	0.72 ± 0.37^b^	1.35 ± 0.28^b^	1.46 ± 0.71	1.23 ± 0.97	1.33 ± 0.38^a^	1.38 ± 0.65
**Material Properties**								
Ultimate Stress (MPa)	36.44 ± 11.7	31.71 ± 5.85	19.35 ± 8.92^b^	30.12 ± 6.38	42.45 ± 15.0	32.40 ± 14.4	33.38 ± 8.02^a^	28.31 ± 8.13^b^
Young’s Modulus (MPa)	276.1 ± 89.6	168.3 ± 36.9^b^	118.5 ± 30.1^b^	167.3 ± 53.1^b^	279.6 ± 95.9	180.2 ± 59.1^b^	191.3 ± 35.0^a,b^	137.5 ± 32.3^b^
Energy to Failure (N-mm/mm^3^)	3.25 ± 0.74	3.59 ± 1.01	2.03 ± 1.24	3.44 ± 0.95	4.77 ± 3.04	3.74 ± 3.06	3.58 ± 1.63	3.60 ± 1.42

^a^Indicates significant differences from WT control at corresponding time point (p < 0.05).

^b^Indicates significant differences from uninjured (p < 0.05).

There were no remarkable effects of PAI-1 deficiency on the viscoelastic stress relaxation response of uninjured or injured tendons. Except for an increase in the stress relaxation percentage in WT tendons at 14 dpi compared to uninjured tendons and the PAI-1 KO injured tendon at the same time point, there were no significant differences attributed to PAI-1 deficiency or time following injury in the stress relaxation percentage, the viscous dissipation parameter C, and the early and late relaxation time constants (τ_1_, and τ_2_, respectively) of the injured tendons.

PAI-1 deficiency did not significantly affect the structural tensile failure properties in uninjured tendons (Table 1). Moreover, at 14 and 28 dpi the maximum force, stiffness, and work to failure of the injured WT flexor tendons were significantly lower than uninjured counterparts. At 56 dpi, stiffness and work to failure of injured WT flexor tendons were significantly lower than uninjured counterparts. In contrast, the stiffness of the injured PAI-1 KO flexor tendons at 28 and 56 dpi and maximum force at 14 dpi were significantly reduced compared to uninjured counterparts. At 28 dpi, however, the maximum force at failure, stiffness, and work to failure were higher in PAI-1 KO compared to WT mice.

PAI-1 deficiency did not significantly affect the material tensile failure properties in uninjured tendons (Table 1). Both the maximum stress and elastic modulus remained significantly decreased over time after injury in both WT and PAI-1 KO flexor tendons compared to uninjured controls. There were no significant differences attributed to PAI-1 deficiency in the energy to failure at any time point. At 28 dpi, however, the maximum stress at failure (ultimate strength) and elastic modulus were significantly higher in PAI-1 KO compared to WT mice. Collectively, these data suggest that PAI-1 deficiency accelerates recovery of tensile structural properties and does not adversely affect the tensile material properties following injury.

### PAI-1 deficiency reduces expression of profibrotic genes and increases early expression of *Mmp2*

**Figure 4:**
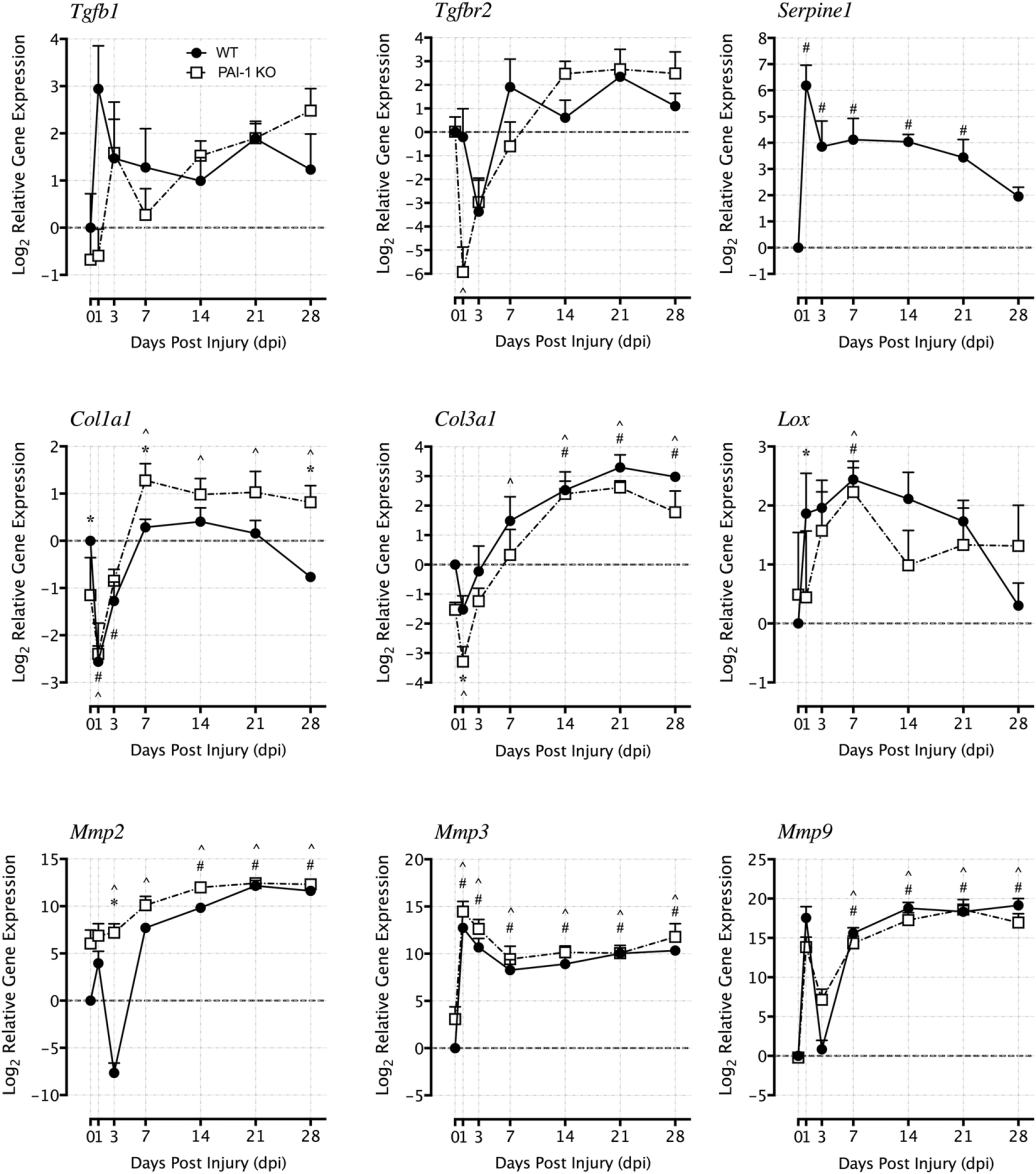
Loss of PAI-1 gene (Serpine1) in the KO flexor tendons led to early downregulation *Tgfb1* & *Tgfbr2* expression with no adverse effects on ECM gene expression and increased early expression of *Mmp2* in comparison to injured WT flexor tendons. Gene expression analysis was performed at 1, 3, 7, 14, 21, and 28 dpi using rtPCR TaqMan Low Density Array (TLDA) designed for fibrosis-related genes. Gene expression levels were normalized to beta-actin as a house keeping gene and then plotted on a log_2_ scale relative to day 1 gene expression levels in the WT flexor tendons. Data are expressed as mean ± standard deviation (SD). Asterisks (*) indicate significant differences between WT and PAI-1 KO (p < 0.05). Pound symbols (^#^) indicate significant differences between uninjured and injured in WT (p < 0.05). Caret symbols (^) indicate significant differences between uninjured and injured in PAI-1 KO (p < 0.05).

Expression of genes associated with tendon fibrosis, remodeling, and signaling (Supplementary Table S2) were evaluated over time using real-time RT-PCR up to 28 dpi. Compared to uninjured tendons, the expression of profibrotic genes changed over time following injury. For example, increased expression of *Tgfb1*, *Serpine1*, and *Lox* was observed throughout the healing time course in WT tendons, while the expression of *Tgfbr2* and *Col3a1* were reduced at early time points but increased after 7 dpi (Fig. 4). The temporal expression of these genes in the PAI-1 KO tendons after injury paralleled the trends observed in the WT tendons. However, the expression levels of *Tgfb1*, *Col3a1* and *Lox* were significantly reduced in PAI-1 KO tendons at 1 dpi compared to WT tendons, and as expected, *Serpine1* gene expression was undetectable in the PAI-1 KO throughout the healing time course. While gene expression of *Col1a1* was significantly reduced in both WT and PAI-1 KO tendons at 1 dpi compared to their uninjured counterparts, *Col1a1* expression increased in injured PAI-1 KO tendons compared to WT tendon at 7 dpi and up to 28 dpi. Expression of remodeling genes *Mmp2*, *Mmp3* and *Mmp9* significantly changed over time following injury in WT tendons. These changes in general exhibited increasing trends, except for sharp reductions at 3 dpi in the expression of *Mmp2* and *Mmp9*. The temporal gene expression of these proteinases in the injured PAI-1 KO tendons were in line with the trends observed in WT tendons. However, PAI-1 deficiency resulted in increased expression of *Mmp2* at early time points compared to WT tendons, with significant differences observed at 3 dpi.

### PAI-1 deficiency enhances MMP activity in the early stages of tendon healing

**Figure 5:**
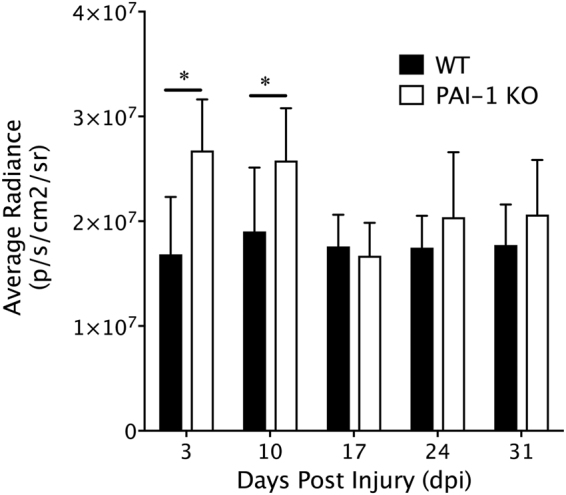
MMP activity is enhanced early after deep digital flexor tendon injury in the PAI-1 KO mice compared to WT. *In vivo* matrix-metalloprotease (MMP) activity, measured longitudinally using fluorescent IVIS imaging following IV delivery of MMPSense 750FAST and quantified in region of interest encompassing the injured paw as photon counts per second per unit area. MMP imaging was performed on days 3, 10, 17, 24, and 31 post injury. Data are presented as mean ± standard deviation. Asterisks indicate significant differences between WT and PAI-1 KO (p < 0.05).

To determine whether PAI-1 influences MMP activity *in vivo*, MMPSense 750 FAST was introduced via tail injections into WT and PAI-1 KO mice 24 hours prior to molecular epifluorescent imaging on the IVIS Spectrum at multiple time-points after the flexor tendon partial laceration injury. MMPSense 750 FAST is fluorescent only after cleavage by MMP 2, 3, 7, 9, 12, and 13, and is used for assessment of pathology-related MMP activity *in vitro* and *in vivo*^25^. Longitudinal assessment at the site of injury demonstrates that PAI-1 deficiency induces significant increases in MMP activity at 3- and 10-dpi. However, protease activity in WT and PAI-1 KO mice were equivalent by 14 dpi and thereafter (Fig. 5).

### siRNA inhibition of *Serpine1* reverses the protease-suppressive effects of TGF-β1 *in vitro*

**Figure 6:**
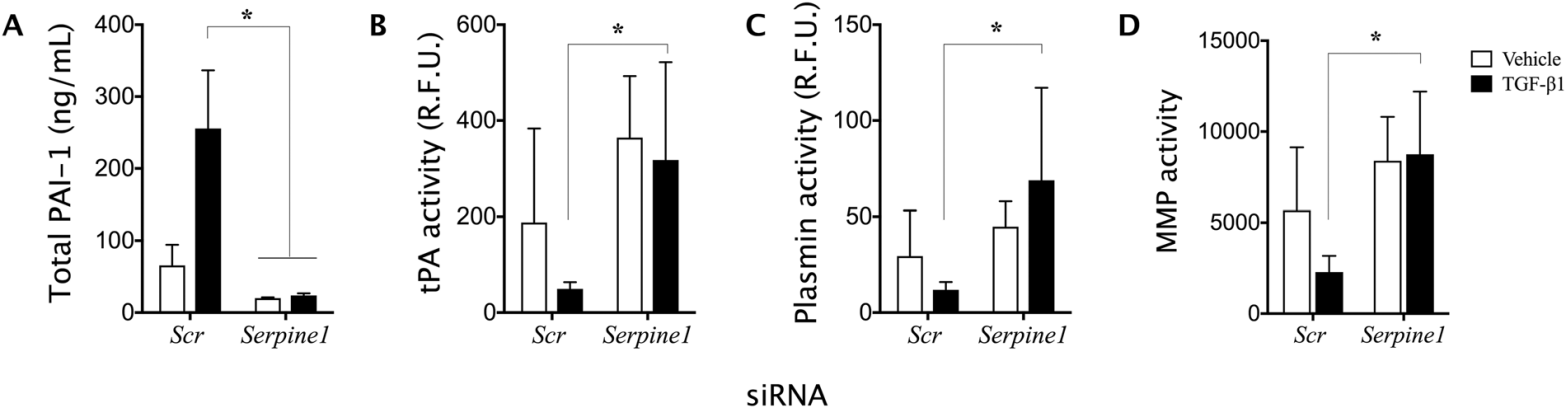
siRNA knockdown of *Serpine1* abrogates TGF-β1 suppression of tPA, plasmin and MMP activity in primary WT tendon cultures. Primary flexor tendon cells from WT mice transfected with scrambled non-targeting siRNA (siRNA^*Scr*^) or siRNA targeting Serpine1 (siRNA^*Serpine1*^) were treated with TGF-β1, tPA and plasminogen for 48 hours. The culture media was then collected and assessed for (**A**) total secreted PAI-1 protein levels and the proteolytic activity of (**B**) tissue plasminogen activator (tPA), (**C**) plasmin, and (**D**) MMPs using fluorescent substrate (FRET) assays. Data are presented as mean ± standard deviation. Asterisks indicate significant differences between siRNA^*Scr*^ and siRNA^*Serpine1*^ (p < 0.05).

Following the comprehensive characterization of flexor tendon healing in the PAI-1 KO mice, we hypothesized that therapeutic inhibition of PAI-1 using siRNA approaches will reverse the inhibitory effects of TGF-β1 on MMP activity in WT tendons. To test this hypothesis *in vitro*, WT flexor tendon cells cultured on collagen-coated substrates were transfected with *Serpine1*-targeting siRNA (siRNA^*Serpine1*^) and non-targeting (scrambled) siRNA (siRNA^*Scr*^), and then treated with TGF-β1, plasminogen and tPA. Protease activity was assessed in the culture media 48 hours later as previously described^11^. Upon treatment with TGF-β1, PAI-1 protein levels in the media significantly increased in cell cultures transfected with siRNA^*Scr*^, but this increase was abrogated in cultures transfected with siRNA^*Serpine1*^ (Fig. 6A). Furthermore, the activity of tPA, plasmin, and MMPs were suppressed significantly by TGF-β1 in cell cultures transfected with siRNA^*Scr*^ (*P* < 0.05), but not in cultures transfected with siRNA^*Serpine1*^ (Fig. 6B–D). These data provide evidence that siRNA inhibition of PAI-1 rescues protease activity despite TGF-β1 signaling and can have therapeutic implications in the treatment of flexor tendon adhesions.

### Nanoparticle-complexed siRNA inhibition of *Serpine1* increases MMP activity in injured tendons

**Figure 7:**
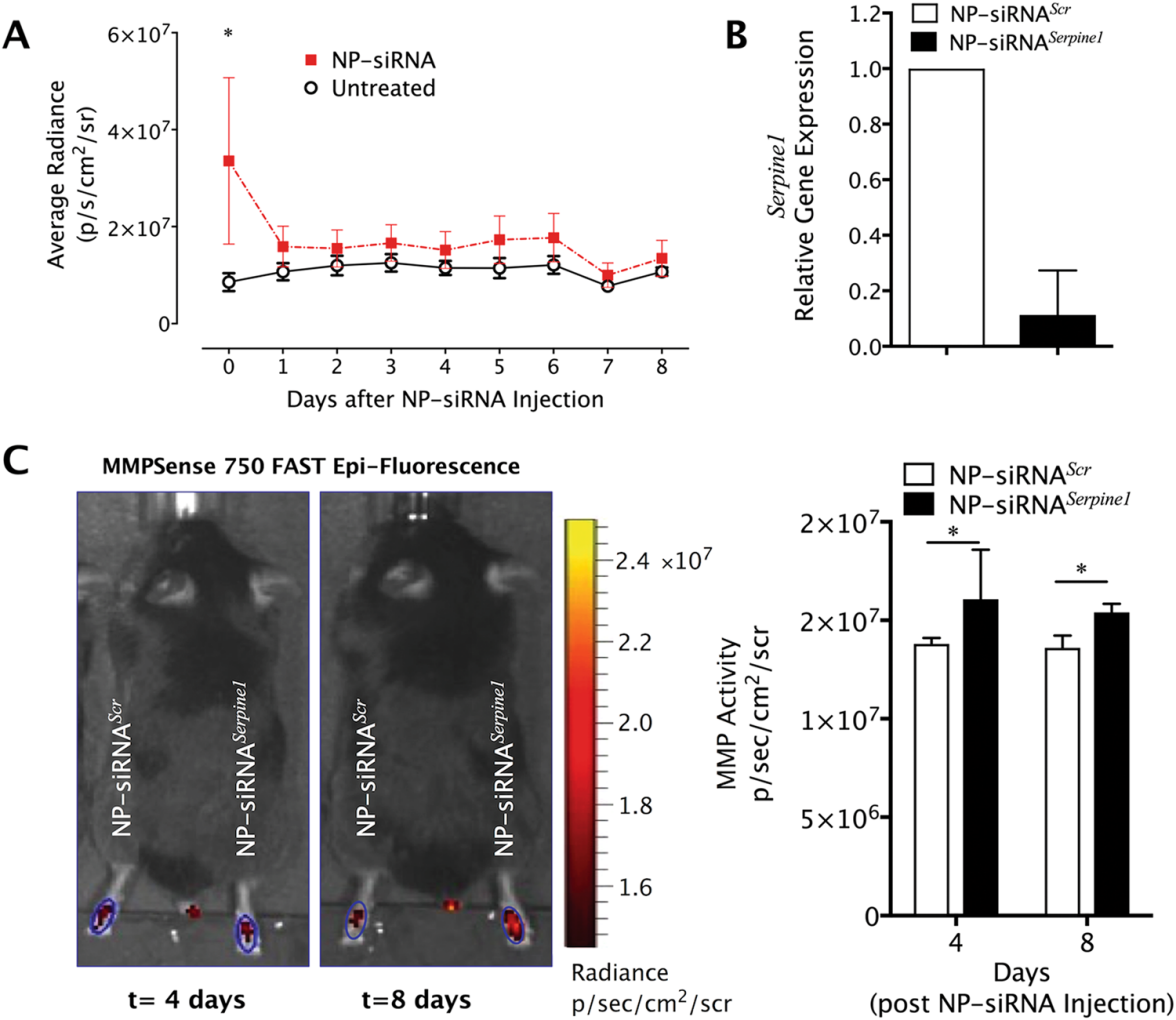
Nanoparticle-mediated delivery of siRNA localizes to the injury site, substantially clears within 24 hrs, but efficiently silences the target gene (*Serpine1*) and enhances MMP activity in the tendon repair tissue. WT mice (n = 3 per treatment) received the standardized partial laceration of the FDP tendon in zone II of the third digit of each hind paw. The mice were then allowed to heal for 7 days, at which time the injured digits were injected with 5 μl of NPs complexed with Cy3-labeled NP-siRNA^*Scr*^ and NP-siRNA^*Serpine1*^ at a dose of 100 pmol/μl. (**A**) NP-siRNA retention was measured over 8 days using IVIS Spectrum and quantified in region of interest encompassing the injected paw as photon counts per second per unit area. (**B**) *Serpine1* gene expression levels were measured using real-time RT-PCR from repair tissue in the injured digit, normalized to beta-actin as a house keeping gene and expressed relative to the NP-siRNA^*Scr*^ treatment. (**C**) MMP activity was longitudinally measured using MMPSense 750 FAST reagent on days 4 and 8 post-injection using IVIS Spectrum and quantified in region of interest encompassing the injured paw as photon counts per second per unit area. Data are presented as mean ± standard deviation. Asterisks indicate significant differences between siRNA^*Scr*^ and siRNA^*Serpine1*^ (p < 0.05).

We sought to demonstrate that siRNA inhibition of *Serpine1* can enhance MMP activity *in vivo* following flexor tendon injury. To overcome challenges associated with siRNA delivery and cellular uptake *in vivo*, we utilized siRNA-complexed with cationic nanoparticles (NP) (NP-siRNA) with pH-responsive endosomolytic behavior to provide efficient intracellular delivery of siRNA *in vivo*, as previously described^26^. To first evaluate delivery and retention kinetics, Cy3-labeled NP-siRNA^*Scr*^ injected in the injured digits of the hind paws of C57Bl/6 J mice at 7 dpi and were tracked longitudinally using IVIS Spectrum imaging, demonstrating that while NP-mediated delivery of siRNA localizes it to the injury site, the fluorescent siRNA signal dissipates substantially after 24 hrs (Fig. 7A). Despite the rapid clearance, *Serpine1* gene expression in NP-siRNA^*Serpine1*^ treated repair tissue was nevertheless reduced by ~70% compared to non-targeting scrambled controls at 72 hours post-injection (Fig. 7B). Furthermore, MMP activity was significantly increased by 10–15% in the NP-siRNA^Serpine1^ treated paws, compared to NP-siRNA^*Scr*^ treated controls on both days 4 and 8 post-NP treatment (Fig. 7C).

## Discussion

Surgical repair of flexor tendon injuries, especially in Zone II of the hand, is often complicated by fibrotic adhesions and can be prone to re-rupture, despite improvements in surgical and suturing techniques and the development of controlled motion rehabilitation protocols^27^. Currently, there exists no pharmacological or biological therapies that can reduce fibrosis and adhesions without compromising repair strength. In this study, we identified PAI-1 as a druggable biological target, which is associated with fibrotic adhesions in flexor tendon healing. Robustly induced by TGF-β1, PAI-1 is a serine protease inhibitor (serpin), which primarily functions to irreversibly inhibit tissue and urokinase plasminogen activator (tPA and uPA, respectively), the activators of plasminogen and hence fibrinolysis. As such PAI-1 is known to regulate the degradation and remodeling of the ECM during wound healing by controlling plasmin and plasmin-mediated MMP activity^11^. Previous studies have evaluated the role of PAI-1 in the etiology of several pathologies, including major organ fibrosis^17^ and cancer^28^.

Since *Mmp2* was the only gene observed to be differentially expressed between WT and PAI-1 KO tendons, it is reasonable to conclude that it is contributing to the observed PAI-1 deficiency effects. However, it has been previously demonstrated that treating tenocytes in collagen gel *in vitro* model of tendon with increasing doses of TGF-β1 results in no remarkable effects on the expression of *Mmp2*, *Mmp3*, and *Mmp14*, but significantly decreases *Mmp16* in a dose-dependent effect^7^. Therefore, it is quite possible that other MMPs, including MMP-16 (also known as MT3-MMP) may contribute to the observed effects of PAI-1 KO downstream of TGF-β1. Interestingly, MT-MMPs (MMP-14 and MMP-16) have been reported to play pivotal roles in cancer cell migration and metastasis. However, not much is known about the MT-MMP’s role in fibrotic tendon healing, which warrants future investigations.

In humans, PAI-1 deficiency is a rare autosomal recessive hereditary disorder, wherein heterozygotes are asymptomatic while homozygotes with complete PAI-1 deficiency are prone to hemorrhage in association with injury and delayed wound healing^29,30^. In mice, however, the effects of PAI-1 deficiency manifest differently. Homozygous PAI-1 KO mice are viable and undergo normal development. While they may exhibit a mild hyperfibrinolytic phenotype, mice are not susceptible to bleeding disorders and hemostasis is normal^31^. PAI-1 KO mice are protected from the collagen accumulation associated with models of cutaneous^12^, renal^32^, hepatic^33^, pulmonary^34^ and cardiac^35^ fibrosis, while transgenic PAI-1 overexpressing mice are more prone to fibrotic pathologies^36^. Furthermore, PAI-1 KO mice heal with no abdominal adhesions after partial hepatectomy, compared to wild type mice, which heal with significant abdominal adhesions^37^. Interestingly, a recent literature survey has identified PAI-1 polymorphisms among predisposing factors to post-operative adhesion development in intra-abdominal and pelvic surgery in humans^38^. Taken together, these reports and our own data in Figs 1 and 2 provide strong rationale to evaluate the role of PAI-1 as a critical mediator of fibrotic adhesions following flexor tendon injuries in mice, which to the best of our knowledge has not been reported previously. Our therapeutic approach uses localized and transient PAI-1 inhibition with the delivery of siRNA specifically to avoid systemic complications, which might be concerning in humans.

PAI-1 satisfies numerous criteria for a drug target^39^. First, findings in this study suggest that PAI-1 inhibition can be disease-modifying (i.e. reduce adhesions) without the biomechanically deleterious effects previously reported for direct inhibitors of upstream regulators of matrix deposition and turnover such as TGF-β1 and its canonical second messengers (Smads)^3,4,40^. Specifically, our findings demonstrate that in a mouse model of genetic PAI-1 deficiency, the intrasynovial flexor tendon healing is characterized by improved tissue morphology, reduced fibrotic adhesions, and increased MMP activity without compromising the tissue biomechanics. Second, the 3D crystal structure of the latent^41^ and active inhibitory forms of PAI-1^42^ have been modeled. This enables mechanistic druggability assessment via characterization of the molecular interactions of chemical compounds and biologics (e.g. small molecule inhibitors or neutralizing antibodies) with specific sites in the 3D structure of PAI-1 and predict their inhibitory or proteolytic effects^19,43^. Third, PAI-1 effects on fibrinolysis and protease activity can be quantified, as we have previously demonstrated^11^, using assays adaptable to high throughput screens, which can be used in 3D microphysiological models of human tendons for novel drug discovery^44^. Fourth, in the context of flexor tendon repair, functional biomarkers such as passive and active flexion of digit joints can offer unmistakable monitoring of therapeutic efficacy. Finally, local modulation of PAI-1 under physiological conditions (i.e. post-surgical repair) at the injury site is not likely to produce any systemic side effects related to excessive or life-threatening hemorrhage since the treatment effects will be short-lived, and global PAI-1 deficiency lethality is rare and only associated with concomitant injury.

There are three approaches or classes of drugs to inhibit biological targets^39^. These include small molecular weight chemical compound (SMOL) inhibitors^20,45,46^, biologics (antibodies or other proteins that induce conformational changes to alter target function or favor the transition from active to latent forms)^3,5,47–50^ and nucleic acids (acting through RNA interference mechanisms)^40,51^. Regardless of drug class, the route of administration is a critical factor in designing molecular therapeutics. Systemic delivery of SMOL inhibitors or biologics for the treatment of major organ fibrotic pathologies might be warranted, but for focal trauma such as tendon adhesions or even abdominal adhesions following general surgery, the challenges associated with pharmacokinetics of systemic delivery^52^ can be avoided using localized delivery approaches.

In this study, we hypothesized that RNA interference (RNAi) can potentially overcome many of these difficulties and can be designed for localized delivery to transiently knockdown the expression of *Serpine1*, the gene coding for PAI-1. The feasibility and efficacy of silencing *Serpine1* using siRNA approaches have shown beneficial antifibrotic effects in the treatment of pulmonary fibrosis^53^. A major limiting factor to siRNA delivery is that most current delivery approaches (polymers and lipids) are not optimized to enable endosomal escape of siRNA into the cytoplasm, where they can then associate with the RNA-induced silencing complex, or RISC, to direct the degradation of the target mRNA. To address these delivery problems, we have developed and adapted diblock copolymer nanoparticles (NP) specifically designed with cationic corona blocks of dimethylaminoethyl methacrylate (DMAEMA) to complex with and protect the siRNA, and pH-responsive hydrophobic core blocks of DMAEMA, butyl methacrylate (BMA), and propylacrylic acid (PAA), which mediate endosomal escape upon protonation when endosomes acidify as they mature into lysosomes^22,54–57^. We have recently evaluated the effects of synthesis variables of this NP-delivery system and demonstrated that the pH-responsive hydrophobic core design is critical for cytocompatiblity and efficient gene silencing in mouse and human musculoskeletal cells including tendon fibroblast^22^. Findings reported herein demonstrated that NP-mediated siRNA knockdown of *Serpine1* in tenocytes rescues plasmin-mediated MMP activity despite treatment with TGF-β1 *in vitro*. We also demonstrated that NP-siRNA delivery significantly knocks down *Serpine1* gene expression at the site of tendon injury *in vivo*, despite rapid clearance of the NP-siRNA.

Furthermore, the therapeutic consequences of inhibition of *Serpine1* gene expression were evident by the increased MMP activity, which paralleled the effects observed in the PAI-1 deficient mice. These results corroborate previous findings in which this NP-siRNA technology was employed to protect mouse salivary glands (SG) from radiation-induced damage^21^. In these studies, our pH-responsive nanoparticles complexed with siRNAs targeting *protein kinase c delta (PKC∆)*, a gene associated with radiation-induced gland senescence, apoptosis, and, ultimately, hyposalvation, were retroductally injected into mouse submandibular glands (SMG), and resulted in efficient knockdown of the target and improved salivary secretion, compared to scrambled siRNA treated controls^21^, further providing validation to this approach as a potential therapy. This NP-siRNA technology has also been successfully employed for controlled release^26^, wherein siRNA targeting WW domain-containing E3 ubiquitin protein ligase 1 (Wwp1) was complexed with the NPs and encapsulated in a PEG hydrogel, and resulted in sustained inhibition of Wwp1 for >10 days, compared to the typical 2 day inhibition window achieved with bolus NP-siRNA delivery. Increasing the duration of target gene inhibition of Wwp1 with a single implant of the hybrid NP/hydrogel system resulted in improved bone fracture healing, providing evidence for the tenability of the system for long-term knockdown based on therapeutic needs^26^.

There are some limitations to the current work. In evaluating the adhesions, histomorphometric outcomes were adapted from Wong *et al*.^58^. These outcomes were further optimized by developing techniques that reduced tissue shrinkage during histology processing. Future work would need to utilize functional biomechanical tests to assess the gliding resistance and flexion function of the injured digits, as we have previously published with a different flexor tendon injury model^4,48^. In this study we have developed and evaluated a novel zone II tendon injury in the central digit of the mouse hind paw. The biomechanics of gliding in the much smaller tissue in the single digit is significantly different than the previously published models^4,48^, and therefore has not allowed reliable and reproducible measurement of gliding resistance using the old technique. Furthermore, our therapeutic studies were proof-of-concept experiments and limited to assessment of *Serpine1* gene knockdown and subsequent effects on MMP activity *in vitro* and *in vivo*. Future studies will aim to validate the current findings and methodically assess the cytotoxicity and functional efficacy of NP-siRNA mediated *Serpine1* knockdown on tendon healing *in vivo*.

Finally, while the current study hypothesizes that PAI-1 inhibition of MMP activity is the underlying cause of adhesions, it is possible that PAI-1 exerts its profibrotic effects via alternative, or possibly synergistic cellular mechanisms. It is possible that the increased MMP activity in the PAI-1 KO led to increased cellular infiltration in the injured tendons compared to WT. However, we did not directly track cellular infiltration. It is also possible that there are PAI-1 deficiency-related differences in cell cycle and potentially senescence. PAI-1 has been well described as a mediator and driver of cellular senescence^46,59–62^ and a member of the senescence-messaging secretome (SMS). Given this cited empirical evidence, the potential cellular mechanism governing the increased cell number and revitalization of the flexor tendon in the PAI-1 KO compared to the C57BL/6 J mice could be attributed to perturbations in cell cycle and the inhibition of apoptosis^62^, which should be investigated in future studies. Interestingly, while cellular senescence has been implicated in fibrotic pathologies^63–65^, the role of PAI-1 in senescence-mediated mechanisms of fibrosis is yet to be explored and should be an area of future investigation.

In conclusion, this study demonstrated that genetic and therapeutic disruption of PAI-1 results in reduced flexor tendon adhesions by tuning MMP activity without compromising the tensile loading biomechanics of the healing tissue. Future preclinical studies of safety and efficacy will be crucial in validating these results and demonstrating that *Serpine1*-targeting NP-siRNA can be an effective treatment for adhesions in flexor tendon surgery and possibly other surgical contexts.

## Materials and Methods

### Animal Care and Tendon Surgery

All animal procedures were conducted in compliance with protocols approved by the University of Rochester Committee on Animal Research (UCAR). The mouse strains utilized in this study were C57Bl/6 J (WT) and B6.129S2-Serpine1^tm1Mlg^/J (PAI-1 KO) mice (Jackson Laboratory). Eight to twelve weeks old male mice from each strain were randomized into experimental groups as per Supplementary Table S1. The mouse surgery protocol involves partial laceration of the deep digital flexor tendon of the 3rd digit of the hind paw as described in Supplementary Information.

### Histology and Immunohistochemistry

Hind paws were severed near the tarsal bones and prepared for histology using routine techniques. For immunohistochemistry tissues were stained with anti-TGF-β1 (1:200, Santa Cruz sc-146) and anti-PAI-1 (1:100, Santa Cruz sc-8979) overnight at 4 °C. For histomorphometry, sections collected between the metatarsophalangeal joint and the A3 pulley of each sample were stained with Mayer’s Hematoxylin and cover slipped (Faramount Aqueous Mounting Medium, Dako, #S3025; Supplementary Figure S2). Magnified micrographs were measured with Image J to determine the volume of the healing tendons, surface area of adhesions, and the gliding synovial space surrounding the tendon.

### Mechanical Testing

For the tensile viscoelastic and failure mechanical testing, the tendons was isolated, measured, and tested in tension in saline according to standard protocols that mainly involved ten cycles of 1% strain preconditioning, followed by a stress relaxation test at 5% strain for 10 minutes, and finally a displacement-controlled ramp to failure at 0.05 mm/sec, as detailed in Supplementary Information (Supplementary Figure S4).

### RNA Extraction and RT-PCR analysis

Tendon tissue from the injury site was harvested for RNA extraction and gene expression analysis were performed by the URMC genomics core. Gene expression was analyzed using real-time reverse-transcriptase polymerase chain reaction (qPCR) TaqMan Low Density Array (TLDA) card for fibrosis, remodeling, and signaling genes of interest (Supplementary Table S2).

### *In Vivo* MMP activity

*In vivo* matrix-metalloprotease (MMP) activity was measured longitudinally using live fluorescent IVIS Spectrum imaging 24 hours following intravenous delivery of MMPSense 750FAST (Perkin Elmer, #NEV10168) (Ex/Em filter 745 nm/800 nm). MMP imaging was performed on anesthetized mice with the dorsal aspect of the foot facing the epifluorescence detectors. Regions of interest (ROI) were placed over the feet to confine the quantification of MMP activity in the lacerated tendons and surrounding tissues. The fluorescence intensities were analyzed by IVIS^®^ Living Image software (Perkin Elmer).

### *In vitro* siRNA delivery

Flexor tendon tenocytes were isolated from 6-week old C57BL/6 male mice and cultured in 12-well collagen-coated plates as previously described^11^. At Passage 7, the cells were transfected using Lipofectamine RNAiMAX (ThermoFisher, # 13778030) with 30 nM siRNA^*Serpine1*^ (ThermoFisher, Silencer Select Serpine1 siRNA, Cat# 4390771, siRNA ID: s71774) or non-targeting siRNA^*Scr*^ (Silencer Select Negative control #1, 4390844, ThermoFisher) according to the manufacturer’s protocols. Untreated cells received an equal treatment volume of 1 × DPBS. The following day, the cells were treated with fresh culture media with or without 10 ng/mL of TGF-β1 (R&D Systems, #240-B-010), with 20 μg/mL of human glu-plasminogen (Haematologic Technologies, HCPG-0130), and 50 ng/mL of tPA (Technoclone, #TC41072).

### Protease Activity Assays and PAI-1 ELISA

Cell culture media was collected for assessment and quantification of secreted PAI-1 and protease activity as previously described^11^, using commercial kits for total PAI-1secreated into the media (Molecular Innovations, #MPAIKT-TOT), tPA, plasmin, and MMP activity (AnaSpec, #72160 and #72125, #60576-01, respectively).

### Polymer Synthesis, Nanoparticle Assembly, and *In Vivo* delivery

Reversible addition fragmentation chain transfer (RAFT) was used to synthesize diblock copolymers that self-assemble into cationic micelles, as described previously^22^. The corona block is composed of cationic dimethylaminoethyl methacrylate (DMAEMA), while the hydrophobic, pH-responsive core block is composed of DMAEMA, butyl methacrylate (BMA), and propylacrylic acid (PAA). This resulted in a monodispersed nanoparticle suspension with a corona to core ratio of 1.6 and PDI of 1.17, nanoparticle diameter of 31 ± 7 nm, and zeta potential of 14 ± 5 mV, as previously characterized^22^. Nanoparticles were complexed to either target siRNA (NP-siRNA^*Serpine1*^) or a scrambled siRNA (NP-siRNA^*Scr*^). Following the flexor tendon injury in C57Bl/6 J mice, 5 µL of 6.3 µg of NP-siRNA^*Serpine1*^ or NP-siRNA^*Scr*^ labeled with Cy-3 (ThermoFisher, Silencer siRNA labeling kit, AM1632) were subcutaneously injected into the laceration site.

### Statistical analysis

All data were graphed or presented in tables as mean ± SEM, and statistically analyzed with GraphPad Prism 7.0 Software. Significant differences (p < 0.05) for all data were determined using a 2-way ANOVA and Bonferroni-corrected multiple comparison post-tests.

## Electronic supplementary material

Supplementary Information

Supplementary Video
