# Supplementary material for: Serpine1 Knockdown Enhances MMP Activity after Flexor Tendon Injury in Mice: Implications for Adhesions Therapy
Source: Sci Rep. 2018 Apr 11;8:5810. doi: 10.1038/s41598-018-24144-1 (PMC5895578; doi:10.1038/s41598-018-24144-1)
Supplement: Supplementary file 1 — Supplementary Information [file 41598_2018_24144_MOESM1_ESM.pdf]

SUPPLEMENTARY INFORMATION

Serpine1 Knockdown Enhances MMP Activity after Flexor Tendon Injury in Mice: Implications for Adhesions Therapy

Margaret A.T. Freeberg, Youssef Farhat, Anas Easa, Jacob G. Kallenbach, Dominic Malcolm, Mark Buckley, Danielle S.W. Benoit, Hani Awad

Table S1. Experimental Design and Sample Size

Genetic PAI-1 Deficiency Studies (PAI-1 KO vs WT DDFT Partial Laceration Healing)		
**Experimental Group**	**Sample Size**	**Time Points**
Histology and Histomorphometry	n=5-6 per time point per mouse strain[1]	Uninjured controls, 3-, 7-, 14-, 28-, and 56-dpi
Biomechanics	n= 6-9 per time point per mouse strain[2]	Uninjured controls, 14-, 28-, and 56-dpi
RT-PCR	n=3 per time point per mouse strain	Uninjured controls, 1-, 3-, 7-, 14-, 21-, and 28-dpi
MMPSense	n=5 per time point per mouse strain	3-, 10-, 17-, 24-, 31-dpi

Therapeutic siRNA-mediated PAI-1 knockdown		
**Experimental Group**	**Sample Size**	**Time Points**
in vitro siRNA RT-PCR Studies	n=3 per group	48 hours post transfection
in vivo siRNA Studies - NP-siRNA Tracking	n=6 per treatment	0, 1, 2, 3, 4, 5, 6, 7, 8 days post NP-siRNA injection
in vivo siRNA Studies - MMPSense	n=3 per treatment	4 and 8 days post NP-siRNA injection

[1] One WT and one PAI-1 KO samples (14-dpi) were excluded because of mixed labels

[2] Samples were excluded when aberrant Force-displacement could be traced down to inappropriate gripping and slippage

Murine Hind Paw Middle Digit Flexor Tendon Partial Laceration Surgery

All animal procedures were conducted in compliance with protocols approved by the University of Rochester Committee on Animal Research (UCAR). The mouse strains utilized in this study were C57Bl/6J (WT) and B6.129S2-Serpine1tm1Mlg/J (PAI-1 KO) mice (Jackson Laboratory). Eight to twelve weeks old male mice from each strain were randomized into experimental groups as per Supplementary Table S2. The mouse surgery protocol involves partial laceration of the deep digital flexor tendon of the 3rd digit of the hind paw. Surgeries were performed under a stereomicroscope on the hind paws using sterile aseptic techniques. Mice were anesthetized with 60 mg/kg ketamine and 4 mg/kg xylazine. A transverse volar skin incision was made on the middle digit between the metatarsophalangeal and the proximal interphalangeal joints. The subcutaneous tissue and the tendon sheath were carefully dissected to expose the flexor tendons. Then the deep digital flexor tendon was separated from the two peri-located flexor digitorum superficialis tendon strips with blunt end micro-forceps. A transverse cut was made mediolaterally across roughly 50% of the width of the deep digital flexor tendon (Supplementary Video). After arranging the structures of the surgical area to their normal anatomical places, the incision was closed using interrupted sutures (Ethicon Suture, V950G, 9 0). To minimize pain, all mice were administered Animalgesics for Mice extended release Buprenorphine at a dose of 3.25 mg/kg every 48 – 72 hours subcutaneously.

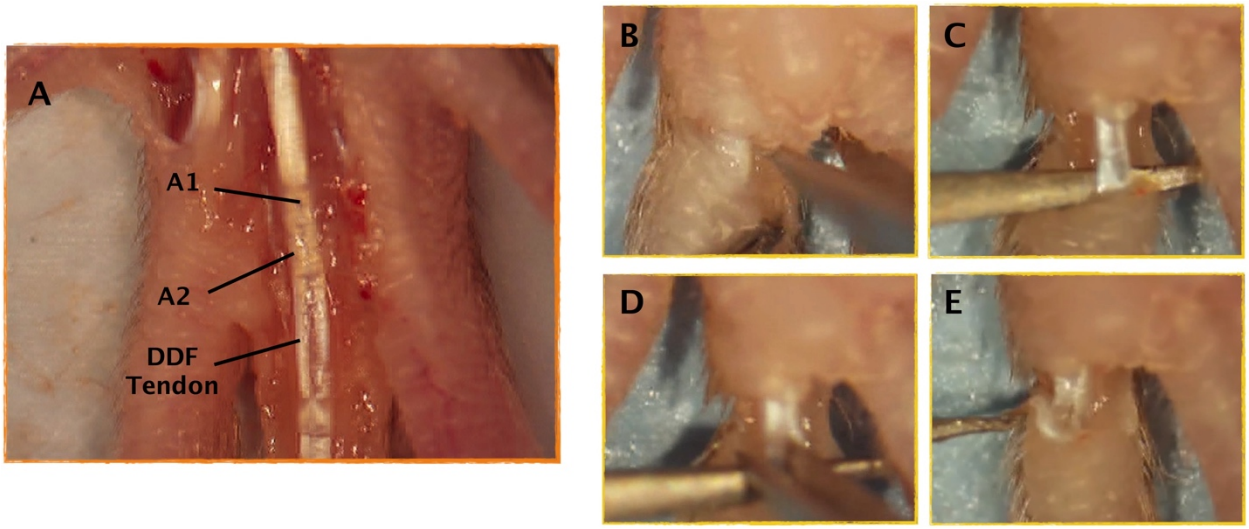

Figure S1. The mouse surgery protocol involves partial laceration of the deep digital flexor (DDF) tendon of the 3rd digit of the hind paw.
(A) A dissected middle digit in the mouse hind paw showing the zone II flexor tendons anatomy.
(B) A transverse volar skin incision is made in zone II of the middle digit.
(C) The deep digital flexor (DDF) tendon is exposed and elevated.
(D,E) A transverse cut is made mediolaterally across roughly half the width of the DDF tendon.

Supplementary Video 1 illustrates the partial laceration surgery

Histology Protocols

Hind paws were severed near the tarsal bones. The tissues were prepared for histology using routine techniques. Briefly, the skin overlying the footpads were scored to enhance 10% neutral buffered formalin (NBF) infiltration into the tissue. Tissues were fixed in 10% NBF for 2 days at room temperature, followed by decalcification in formic acid for 9 days at room temperature, then dehydrated in an ethanol gradient and embedded in paraffin. For immunohistochemistry tissues were cut longitudinally into 5 µm thick sections, washed with PBS and blocked with 1% bovine serum albumin (BSA) in PBS for 30 min. Sections were then incubated with anti-TGF-β (1:200, Santa Cruz sc-146) and anti-PAI-1 (1:100, Santa Cruz sc-8979) overnight at 4°C. Subsequently, after washing with PBS, the sections were incubated with anti-rabbit secondary antibody for 20 min and then with peroxidase substrate, counterstained with hematoxylin and cover slipped. For histomorphometry, 5 µm thick sections collected between the metatarsophalangeal joint and the A3 pulley of each sample and were stained for 5 minutes with Mayer's Hematoxylin and cover slipped (Faramount Aqueous Mounting Medium, Dako, #S3025; Supplementary Figure S2). Magnified micrographs were measured with Image J to determine the volume of the healing tendons, surface area of adhesions, and the gliding synovial space surrounding the tendon.

Most tissue stains, including Hematoxylin/Eosin, Masson's Trichrome, and Alcian Blue/Hematoxylin/Orange G, require ethanol dehydration prior to cover-slipping with an organic solvent based mounting medium (e.g. acrylic resin) to preserve specimen and prevent dyes from fading over time. After carefully observing samples before, during, and after the various steps of several staining protocols, it was determined that ethanol dehydration followed by the mounting with organic medium resulted in notable distortion of tendon and adhesion morphology in mouse digit cross-sections, precluding an accurate quantification of adhesions with histomorphometry. Specifically, tendons shrank considerably, causing them to fracture internally and pull away from the surrounding tissue, destroying significant portions of the tendon-subcutaneous tissue interface (i.e. adhesions) that were originally intact prior to staining (Figure S2, panels A and B).

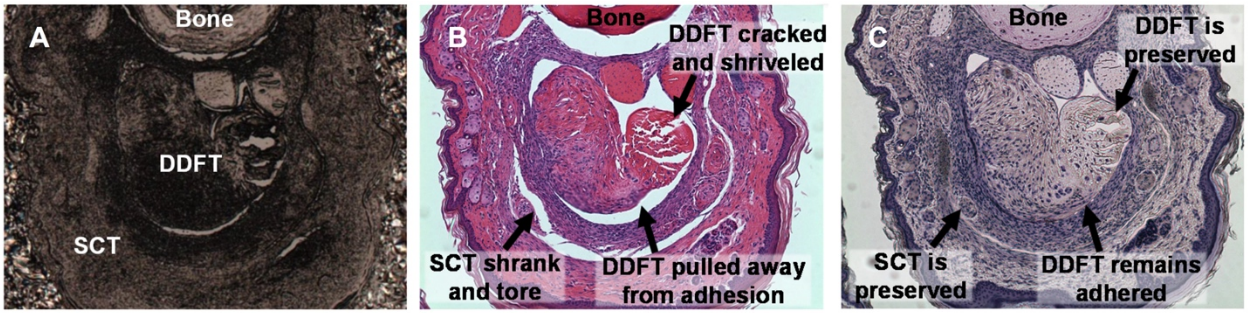

Figure S2. Aqueous-mounting preserved tendon and adhesion morphology, enabling the quantification of adhesions with histomorphometry. (A) Paraffin-embedded digit cross-section prior to deparaffinization and staining. Subcutaneous tissue (SCT) and deep digital flexor tendon (DDFT) are well preserved with minimal sectioning artifacts. (B) Digit cross-section after staining with Hematoxylin and Eosin (H&E), followed by ethanol-dehydration and cover-slipping with an acrylic resin mounting medium. Stained samples have brilliant colors, but various aspects of the DDF tendon and subcutaneous tissue are destroyed due to dehydration-related shrinkage (arrows). (C) Digit cross-section from the same sample after staining with Mayer's Hematoxylin, followed by cover-slipping with an aqueous-mounting medium. Note that the morphology of the DDF tendon and subcutaneous tissue were preserved well (arrows), enabling a more accurate assessment of tendon adhesions.

In order to avoid these artifacts, the axial sections used for histomorphometric analysis were all stained for 5 minutes with Mayer's Hematoxylin and cover-slipped with a water-based mounting medium (Faramount Aqueous Mounting Medium, Dako, #S3025), which allowed for excellent preservation of tissue morphology as well as sufficient staining to distinguish between normal and injured tendons, pulleys, and subcutaneous tissues (Figure S2, panel C).

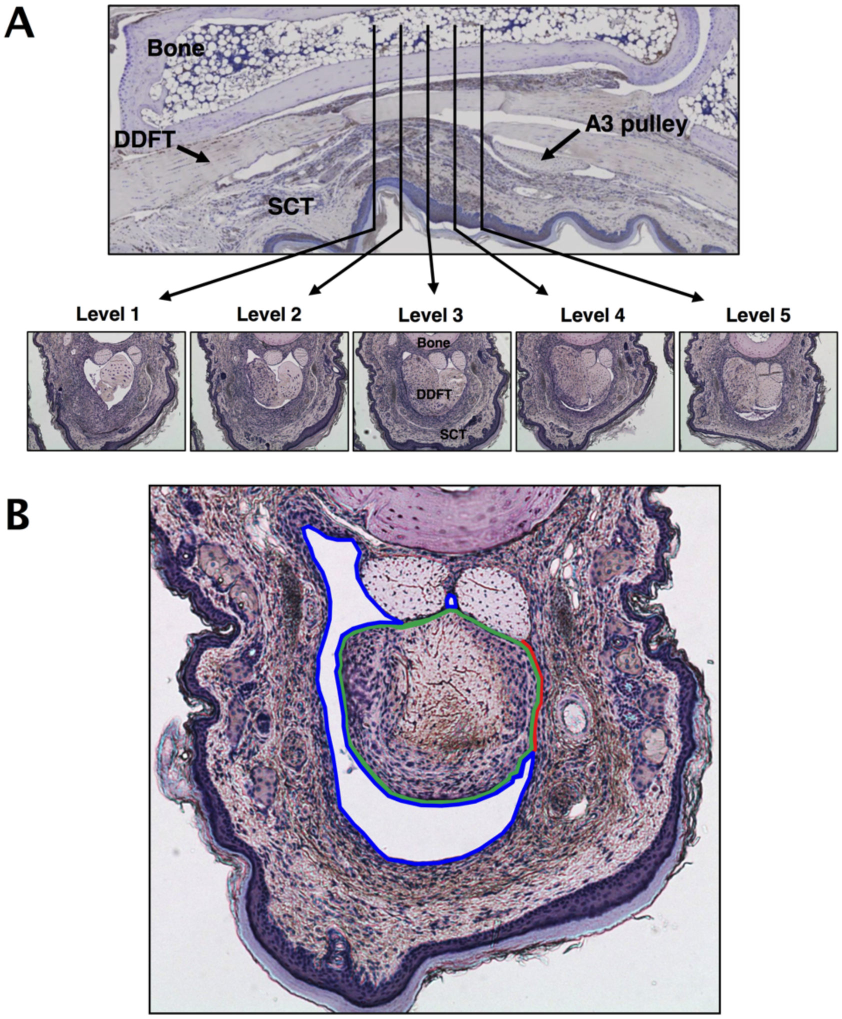

Figure S3. Tissue sampling approach used for histomorphometry for quantitative assessment of adhesions, tendon area, and sheath space. (A) Hematoxylin-stained longitudinal section of a mouse digit two weeks after zone II injury, with the locations of the proximal phalanx bone, deep digital flexor tendon (DDFT), and subcutaneous tissue (SCT) indicated. The five levels chosen for the histomorphometric analysis of each sample were 100 μm apart from one another, and were located immediately proximal to the A3 pulley, as shown. Example micrographs from the five levels of an axially sectioned sample, two weeks after zone II injury, which were used to measure the amount of DDF tendon adhesions, size of the DDF tendon, and sheath space. (B) 10x magnified micrograph of a hematoxylin-stained mouse hind paw digit within zone II, 28 days after partial laceration. Adhesion length (**red**), DDF tendon cross-sectional area (**green**), and sheath space area (**blue**) were manually delineated and measured using ImageJ software. Five serial sections from each sample were delineated in a similar fashion, and measurements were used to compute tendon area and volume, adhesion surface area, and sheath space volume, respectively.

RNA Extraction and RT-PCR Analysis

Tendon tissue from the injury site was harvested for RNA extraction and gene expression analysis were performed by the URMC genomics core. Briefly, tissue was flash frozen in liquid nitrogen and stored at -80□C until time of extraction. Total RNA was isolated from single tendons utilizing Trizol (ThermoFisher, #15596026) extraction methods and a bullet blender for tissue homogenization. Gene expression assessment was performed using real-time reverse-transcriptase polymerase chain reaction (qPCR) TaqMan Low Density Array (TLDA) card for fibrosis, remodeling, and signaling genes of interest (Supplementary Table S2). Gene expression analysis was performed using applied biosystems qPCR analysis modules (ThermoFisher), which utilizes comparative Ct methods (ΔΔCt). Gene expression was normalized to GAPDH and then normalized to uninjured WT gene expression level and plotted on a Log2 scale.

Table S2. TaqMan primers used for qPCR analysis

Gene	Common Name	Assay ID
Acta2	Actin - Alpha 2, smooth muscle, aorta	Mm00725412_s1
Col1a1	Collagen type 1, alpha 1	Mm00801666_g1
Col3a1	Collagen type 3, alpha 1	Mm01254476_m1
Ctgf	Connective tissue growth factor	Mm01192932_g1
EEF1a1	Eukaryotic translation elongation factor 1 alpha 1	Mm01973893_g1
Fbn1	Fibrillin 1	Mm00514908_m1
Fbn2	Fibrillin 2	Mm00515742_m1
Fn1	Fibronectin	Mm01256744_m1
GAPDH	Glyceraldehyde-3-phosphate dehydrogenase	Mm99999915_g1
Il10	Interleukin-10	Mm00439614_m1
Il12	Interleukin-12	Mm00434165_m1
Lox	Lysyl oxidase	Mm00495386_m1
Mhk	Mohawk	Mm00617017_m1
Mmp1a	Matrix metalloproteinase (interstitial collagenase)	Mm00473485_m1
Mmp2	Matrix metalloproteinase (gelatinase)	Mm00439498_m1
Mmp3	Matrix metalloproteinase (Stromelysin)	Mm00440295_m1
Mmp9	Matrix metalloproteinase (gelatinase)	Mm00442991_m1
Plat (tPA)	plasminogen activator, tissue	Mm00476931_m1
Plg	Plasminogen	Mm00447087_m1
Scx	Scleraxis	Mm01205675_m1
Serpine1	Serpin peptidase inhibitor, clade E (plasmiogen activator inhibitor type 1)	Mm00435860_m1
Tgfb1	Transforming growth factor beta 1	Mm01178820_m1
Tgfbr2	transforming growth factor, beta receptor II	Mm00436977_m1

Biomechanical Testing

For the tensile viscoelastic and failure mechanical testing, the tendon was released at the myotendenous junction and dissected along the tibia and tarsal bones, releasing pulleys in the tarsal tunnel. At the tendon pentafurcation in the sole of the foot, the middle (third) flexor tendon was isolated. The tendon-to-bone attachment (enthesis) of the middle digit was left intact for gripping. Tendons were loaded in a custom uniaxial microtester (eXpert 4000 MicroTester, ADMET, Inc., Norwood, MA), and were gripped and superglued in between two stainless steel plates. Tendon width, thickness, and gauge length were measured after preloading protocol to 0.05 N, while the tissue was immersed in a 1x PBS bath and imaged on the microscope (Table S4). Cross sectional area was assumed to be an ellipse. Each sample underwent uniaxial displacement-controlled testing of 1% strain preconditioning, based on total gauge length, for 10-cycles, rested for 5 minutes, followed by loading to 5% strain over 8 seconds and holding for 10 minutes to allow for stress-relaxation, then returned to unloaded gauge length for a 5-minute rest period, and concluded with a displacement-controlled ramp to failure at 0.05 mm/sec (Supplementary Figure S4). Viscoelastic stress-relaxation data were fitted to a quasi-linear viscoelasticity (QLV) model to extrapolate tissue properties. Structural and material properties were determined from the ramp to failure force-displacement and stress-strain curves, respectively. All mechanical testing analysis was completed with a custom Matlab (MathWorks, Natick, Massachusetts) code.

Table S4. Tendon cross-sectional area measurements (mm^2)

	**Uninjured**	**14 dpi**	**28 dpi**	**56 dpi**
**C57Bl6/J**	0.1066 ± 0.012	0.0713 ± 0.0023 [b]	0.07888 ± 0.0026 [b]	0.08806 ± 0.0041 [b]
**PAI-1 KO**	0.07736 ± 0.0033 [a]	0.07482 ± 0.0029	0.08208 ± 0.0050	0.08631 ± 0.0043

[a] indicates significant differences from WT control at corresponding time point ($p<0.05$)
[b] indicates significant differences from uninjured ($p<0.05$)

Quasi-linear viscoelasticity Model Equations

The quasi-linear viscoelasticity (QLV) model developed by Fung [1] describes the stress response of a material as a function of strain history and time. In essence, it decouples the stress response into a nonlinear elastic loading phase and a time-dependent viscoelastic relaxation phase.

The applied strain is defined as:

$$\varepsilon(t) = \begin{cases} \gamma * t \,, t \le t_1 \\ \gamma * t_1 \,, t > t_1 \end{cases} \qquad \text{Eq. 1}$$

Where γ is the nominal strain rate (0.05 strain/8 seconds), and t_1 is the time of the applied nominal strain.

The instantaneous nonlinear elastic response during the loading phase is defined as:

$$\sigma(\varepsilon) = A(e^{B*\varepsilon} - 1) \qquad \text{Eq. 2}$$

where A and B are model parameters whose physical interpretation can be derived by differentiating Eq. 2

$$\frac{\partial\sigma(\varepsilon)}{\partial\varepsilon} = A \times Be^{B*\varepsilon} \qquad \text{Eq. 3}$$

Eq. 3 can be rearranged as

$$\frac{\partial\sigma(\varepsilon)}{\partial\varepsilon} = A \times Be^{B*\varepsilon} + A \times B - A \times B = B(A(e^{B*\varepsilon} - 1) + A \times B$$

Thus, we can write

$$\frac{\partial\sigma(\varepsilon)}{\partial\varepsilon} = B\sigma + A \times B \qquad \text{Eq. 4}$$

Hence, $A \times B$ represent the initial slope of the nonlinear elastic stress-strain curve, while B is related to the rate of change of the slope of the nonlinear elastic stress-strain curve.

The QLV model assumes that the stress-relaxation response (Supplementary Figure S5) following an applied strain can be described by the convolution integral of the reduced relaxation function, G(t). This form of the equation has been used to describe the viscoelastic behavior of tendons and ligaments [2-7].

$$\sigma(t) = \int_0^t G(t-\tau)\frac{d\sigma(\varepsilon)}{d\varepsilon}\frac{d\varepsilon}{d\tau}d\tau \qquad \text{Eq. 5}$$

The relaxation function, G(t), is defined as (Equation 2):

$$G(t) = \frac{1 + C\left[E_1\left(\frac{t}{\tau_2}\right) - E_1\left(\frac{t}{\tau_1}\right)\right]}{1 + Cln\left(\frac{\tau_2}{\tau_1}\right)} \qquad \text{Eq. 6}$$

where C is a dimensionless relaxation index, τ_1 is the early relaxation time constant, τ_2 is the late relaxation time constant, and E_1 is the exponential integral function.

Using a custom Matlab code, the QLV model (Equation 7) was fit to the ramp and relaxation data.

$$\sigma(t) = \frac{AB\gamma}{1 + Cln\left(\frac{\tau_2}{\tau_1}\right)}\int_0^t \left[1 + C\left[E_1\left(\frac{t}{\tau_2}\right) - E_1\left(\frac{t}{\tau_1}\right)\right]\right](e^{B\gamma t})\frac{d\varepsilon}{d\tau}d\tau \qquad \text{Eq. 7}$$

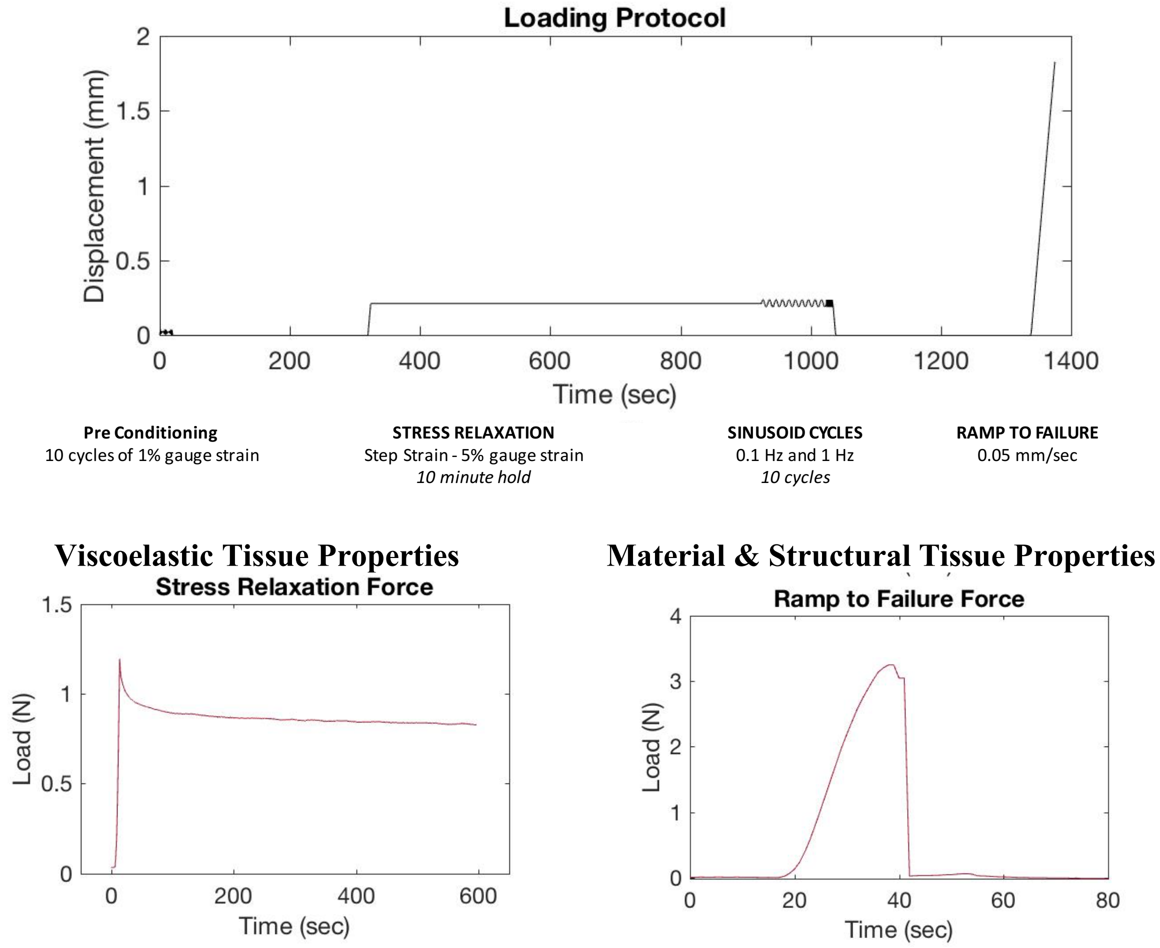

Figure S4. Experimental Biomechanical Testing Protocol. Murine tendons were tested to determine the viscoelastic, material, and structural properties. (A) Tissue loading protocol consisted of 4 phases with rest periods in between. All samples were pre-conditioned with 10 cycles to 1% gauge strain, followed by a stress-relaxation test where a 5% gauge length step-strain was applied and held for 10 minutes, then 10 sinusoidal cycles at 0.1 Hz and 1Hz, and lastly ramp to failure. (B) Representative tissue force during the stress-relaxation test. (C) Representative force-time curve from the ramp to failure test.

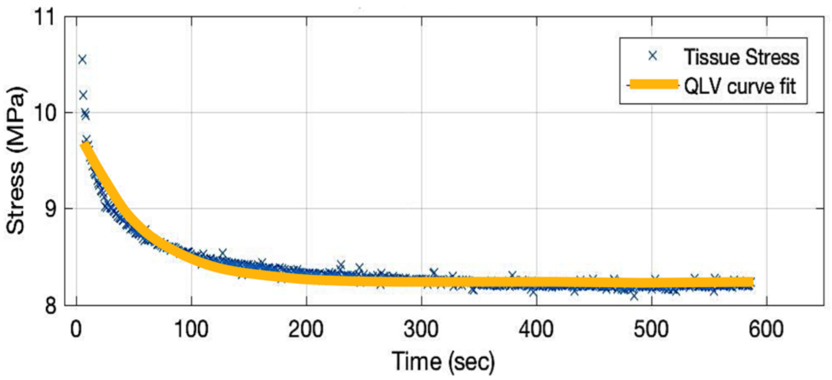

Figure S5. Representative QLV Stress Relaxation Fit. The tissue stress response to the applied step-strain was curve fit to a QLV model using a custom Matlab code.

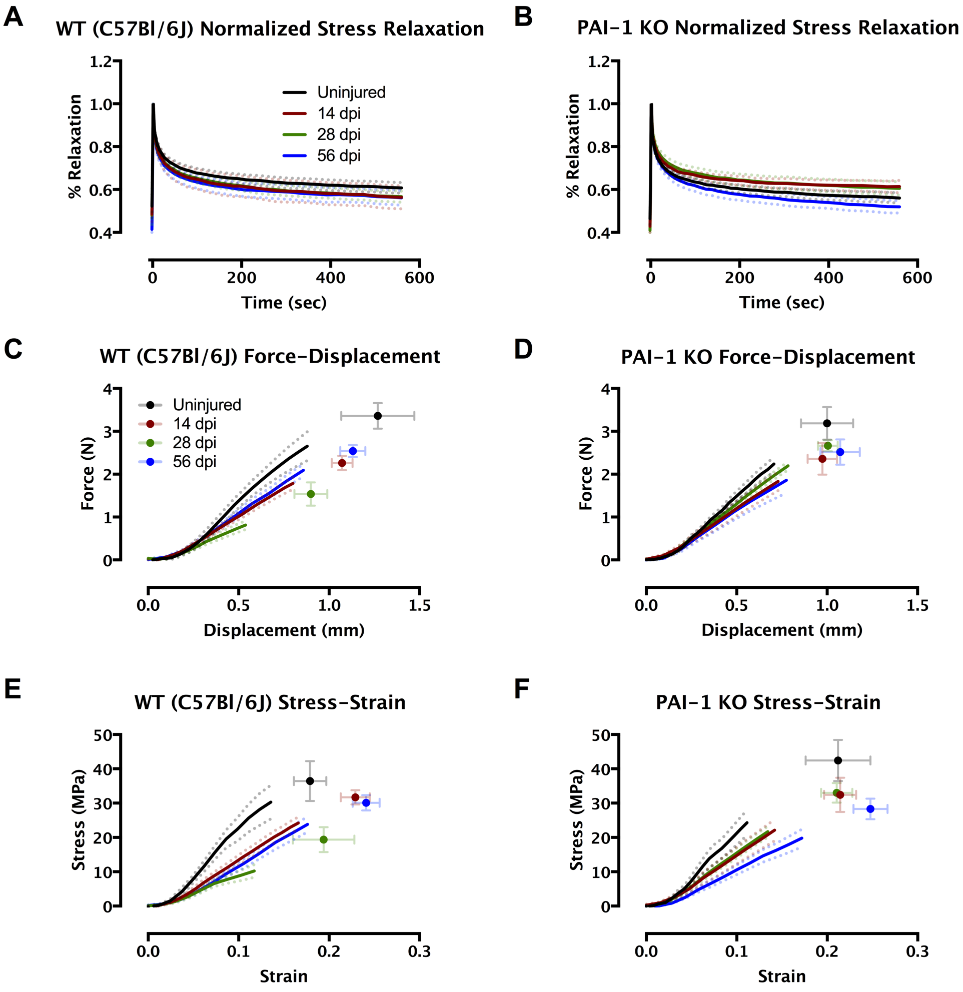

Figure S6. Average Tensile Stress-Relaxation and Displacement-Controlled Tensile Failure Curves. (A & B) Average stress-relaxation curves (± SEM) for both murine strains normalized to the peak stress. (C & D) Average force-displacement curve (± SEM), showing average toe and linear region lines and average failure points per time-point. (E & F) Average stress-strain curves (± SEM) showing average toe and linear region lines and average failure points per time-point.

References

1. Fung, YC (1972). Stress-strain history relations of soft tissues in simple elongation. In: Y.C. Fung, NP, M. Anliker (ed). *Biomechanics: Its Foundations and Objectives*. Prentice Hall: Englewood Cliffs. pp 181-208.
2. Huang, CY, Wang, VM, Flatow, EL, and Mow, VC (2009). Temperature-dependent viscoelastic properties of the human supraspinatus tendon. *J Biomech* **42**: 546-549.
3. Duenwald, SE, Vanderby, R, Jr., and Lakes, RS (2010). Stress relaxation and recovery in tendon and ligament: experiment and modeling. *Biorheology* **47**: 1-14.
4. Criscenti, G, De Maria, C, Sebastiani, E, Tei, M, Placella, G, Speziali, A*, et al.* (2015). Quasi-linear viscoelastic properties of the human medial patello-femoral ligament. *J Biomech* **48**: 4297-4302.
5. Castile, RM, Skelley, NW, Babaei, B, Brophy, RH, and Lake, SP (2016). Microstructural properties and mechanics vary between bundles of the human anterior cruciate ligament during stress-relaxation. *J Biomech* **49**: 87-93.
6. Babaei, B, Velasquez-Mao, AJ, Thomopoulos, S, Elson, EL, Abramowitch, SD, and Genin, GM (2017). Discrete quasi-linear viscoelastic damping analysis of connective tissues, and the biomechanics of stretching. *J Mech Behav Biomed Mater* **69**: 193-202.
7. Woo, SL (1982). Mechanical properties of tendons and ligaments. I. Quasi-static and nonlinear viscoelastic properties. *Biorheology* **19**: 385-396.
